# The Kinesiologist Like a Tailor: The Art of Making a Tailor-Made Physical Activity Plan

**DOI:** 10.3390/jfmk6030076

**Published:** 2021-09-14

**Authors:** Giuseppe Musumeci

**Affiliations:** 1 Department of Biomedical and Biotechnological Sciences, Human, Histology and Movement Science Section, University of Catania, Via S. Sofia n°87, 95123 Catania, Italy; g.musumeci@unict.it; 2 Research Center on Motor Activities (CRAM), University of Catania, Via S. Sofia n°97, 95123 Catania, Italy; 3 Department of Biology, Sbarro Institute for Cancer Research and Molecular Medicine, College of Science and Technology, Temple University, Philadelphia, PA 19122, USA

The skilled work of the tailor comes from ancient and complex art. Precision, care, and attention to the material handled and the subject for which it works. The best tailor takes measurements of everything, neck, shoulders, torso, arms, legs, hands, feet. He takes every human body measure and produces a suit that fits perfectly. With this metaphor, I want to bring attention to the kinesiologist. These two professions have a lot in common. Both of them work with the aid of a tape measure, take anthropometric measurements, create a product tailored to the subject; respectively, the suit made by the tailor, and the corrective exercise made by the kinesiologist.

The kinesiologist uses every valid instrument to measure the human body and study its movement. The term derives from the Greek “kìnesis” for movement, and “logos” for study, and the first to introduce this topic was a Swedish therapist, August Georgii [1]. The kinesiologist accomplishes the anthropometric measurements, whose term means “man” and “measure”, namely the science that measures the human body in its totality or its components. At the same time, a more exciting definition is that of posturometry, defined as a science that measures the weights, lengths, and angles of the human body or parts of it. Over the years, the evolution of this profession has intertwined with multiple areas, gathering tools from every sector such as medicine for the analysis and knowledge of vital parameters, physics for understanding the laws of mechanical physics applied to the human body, gymnastics to understand the objectives of applied physical exercise, and psychology to understand the functioning of the psyche behind the execution of a movement. 

Measuring the length of a limb often comes with measuring its strength as well. Muscle strength is an important test as it allows the evaluation of the general strength of the examined area. The kinesiologist does not deal with diagnosing debilitation; more than anything else, he evaluates muscle tension, the inability to manage a muscle contraction or shortening that can alter the body’s balance, or an asymmetry between the two body sides. The kinesiologist evaluates what has no measures, rebalances what is unstable. 

A slightly different version of the kinesiologist proposed by the chiropractor George J. Goodheart introduced the term “Applied Kinesiology” in 1964. This discipline differs somewhat from the fundamental concept of kinesiology because it interprets the response of muscle tests, weakness, or delayed response as a dysfunction of the nervous system, namely the study of functional neurology [2]. To date, it is a controversial discipline where scientific research has yet to ascertain its possible reliability.

Returning to the tailor origins, the term deriving from the Latin “sartor”, indicates someone who patches or mends textiles. It explains, even more, the work of the kinesiologist who does not limit himself to taking measurements of the subject’s body nor elaborating a specialized gymnastics plan. He deals with patching or mending those movements, postures, exercises, and wrong vices of the human body that the evolution of the species brings with it. The kinesiologist is the figure able to develop a physical exercise plan that aims to manage many chronic diseases that affect not only the musculoskeletal system, but also cardiovascular, respiratory, urinary, digestive, and nervous systems. 

Pedersen and Saltin, in a review [3], reported the evidence of physical exercises prescribed over 26 different chronic diseases. This exciting work highlights not only how the prescription of physical activity is necessary but how the correct execution of a detailed exercise plan for each pathology can lead to improvements as if it were a medicine. The kinesiologist is called upon to do just that: sew a detailed tailored physical activity plan to the patient’s pathologies.

The evolution of the times also requires the evolution of instruments, useful to collect measurements quickly and reliably. Infrared systems, such as 3D cameras and rasterstereography, are becoming increasingly available given their high reliability and ease of measurement, but also to ease the kinesiologist’s job. For example, Wang et al. [4] examined the development of an anthropometric analysis system with a 3D scanner. This system, compared to traditional measurements, gives accurate and consistent results. Although the measurements can be fast and easy, the kinesiologist’s expertise is always required to validate the measurements since digital systems to date can only be configured as auxiliaries for human work, not substitutes.

Like a tailor, being a kinesiologist means observing, measuring, understanding, and studying every detail of the human body, from the body shapes to the movement it performs, to produce the best “dress” based on the lines of each human.
